# DNA-based copy number analysis confirms genomic evolution of PDX models

**DOI:** 10.1038/s41698-022-00268-6

**Published:** 2022-04-28

**Authors:** Anna C. H. Hoge, Michal Getz, Anat Zimmer, Minjeong Ko, Linoy Raz, Rameen Beroukhim, Todd R. Golub, Gavin Ha, Uri Ben-David

**Affiliations:** 1 Public Health Sciences Division, Fred Hutchinson Cancer Research Center, Seattle, WA USA; 2 Department of Human Molecular Genetics and Biochemistry, Faculty of Medicine, Tel Aviv University, Tel Aviv Israel; 3 Broad Institute of Harvard and MIT, Cambridge, MA USA; 4 Dana-Farber Cancer Institute, Boston, MA USA; 5 Harvard Medical School, Boston, MA USA

**Keywords:** Cancer genomics, Cancer models

## Abstract

Genomic evolution of patient-derived xenografts (PDXs) may lead to their gradual divergence away of their tumors of origin. We previously reported the genomic evolution of the copy number (CN) landscapes of PDXs during their engraftment and passaging^1^. However, whether PDX models are highly stable throughout passaging^2^, or can evolve CNAs rapidly^1,3^, remains controversial. Here, we reassess the genomic evolution of PDXs using DNA-based CN profiles. We find strong evidence for genomic evolution in the DNA-based PDX data: a median of ~10% of the genome is differentially altered between matched primary tumors (PTs) and PDXs across cohorts (range, 0% to 73% across all models). In 24% of the matched PT-PDX samples, over a quarter of the genome is differentially affected by CN alterations. Moreover, in matched analyses of PTs and their derived PDXs at multiple passages, later-passage PDXs are significantly less similar to their parental PTs than earlier-passage PDXs, indicative of genomic divergence. We conclude that PDX models indeed evolve throughout their derivation and propagation, and that the phenotypic consequences of this evolution ought to be assessed in order to determine its relevance to the proper application of these valuable cancer models.

Genomic evolution refers to the genetic alteration of the tumor genome over time, including selection or *de novo* acquisition of genetic aberrations, and can occur in the context of clonal expansion due to selection or genetic drift^1^. Copy number alterations (CNAs) are hallmark genomic structural aberrations in many epithelial cancers, and have been shown to associate with tumor progression and treatment resistance^2,3^. The CNA landscapes of patient-derived xenograft (PDX) tumors have been shown to evolve during engraftment and passaging^4–7^, although their interpretation is still being debated, and thus more careful and systematic evaluation is required.

We have previously analyzed copy number (CN) profiles of PDX samples from various passages and cancer types^4^. We found the overall correlation of CN landscapes between PTs and PDXs, and that between related PDXs, to be very high (Pearson’s r = 0.79 for the median CN correlation between PDXs and their respective TCGA tumor type). However, at the individual tumor level, we found that a median of 12.3% of the genome was differentially altered within four passages of PDX models (range, 0% to 59%). Consistent with these results, several recent papers have also reported genomic evolution throughout the engraftment and passaging of PDXs^6,8–16^. The degree of genomic evolution observed in PDXs was similar to that observed by us and others in patient-derived in vitro models (cell lines^17^ and organoids^18^), highlighting the importance of this phenomenon to cancer modeling (reviewed in^19^).

Recently, an international consortium led the assembly of CN profiles for 1,451 samples corresponding to 509 PDX models^5^. CN profiles were evaluated using several methods, including SNP arrays, whole exome (WES) and genome (WGS) sequencing, and RNAseq. To assess the similarity between PTs, early-passage PDXs and later-passage PDXs, the study mostly relied on a Pearson’s correlation analysis of the log_2_(CN ratio) values across the genome, reporting high correlations between the CN profiles of PTs and PDXs (median correlation PT-PDX = 0.950), and between matched pairs of PDXs from different passages (median correlation PDX-PDX = 0.964). Furthermore, the authors reported that the CN profiles remained highly stable throughout in vivo passaging, so that high-passage PDX models represented the CN landscapes of the PTs and of the early-passage PDXs. No recurrent alterations of a specific locus, gene or pathway during PDX engraftment or passaging was reported, which was interpreted as evidence for lack of selection. Based on these results, the authors suggested that the genomic evolution previously observed in PDXs might have been an artifact of inaccurate expression-based CN profiling.

To investigate the apparent discrepancies between the results in these studies, we re-analyzed the CN data from the Woo dataset of 1,451 PDX samples^5^. We initially focused only on DNA-based (SNP arrays, WES, and WGS) CN profiles to avoid any potential issues with expression-based CN inference. In the Woo dataset, this included 33 cohorts of PDX models representing 16 cancer types, each with at least one pair of matched samples that had high-quality DNA-based CN calls^5^. We divided the genome into bins of 1 Mb length, and evaluated the discordance of each bin between PTs and PDXs, using conservative thresholds for ‘discordance’ (Methods). As an alternative approach, we predicted integer CN using ichorCNA^20^, which explicitly accounts for tumor impurity in the primary tumors and potential mouse contamination in the PDX samples, and focused downstream analysis on clonal events to allow for a conservative discordance comparison. For both approaches, we computed the percentage of the genome that showed discordance based on copy number calls, rather than the Pearson’s correlation of the normalized CN values (Supplementary Fig. 1a**;** Methods). Using both approaches, the absolute number of CN alterations and the fraction of the genome affected by them were not significantly different between PTs and their matched PDXs (Supplementary Fig. 1b,c), in line with recent studies^4–6^.

Next, the fraction of the genome discordant between two samples was defined for each PDX model. We also evaluated the number of discordant chromosome-arm CNAs in order to assess the contribution of aneuploidy to copy number evolution. A chromosome arm was defined as discordant if >=75% of the bins within that arm were discordant (Methods). Samples with <5% of the genome affected by CNAs were excluded from the analysis to avoid inflated discordance due to tumor impurity, and only weak correlation was observed between estimated tumor purity and genomic divergence in the remaining samples (Supplementary Fig. 2). The per-sample discordance values were very similar, but not identical, between the two analyses (Pearson’s *r* = 0.7; *p* = 3×10^−60^), demonstrating that they were highly consistent but not completely redundant (Supplementary Fig. 3). The use of copy-number calls to directly compare the CNA landscapes of matched samples is commonly applied in cancer genomics studies to assess CN evolution because the results are readily interpretable, and it allows for a direct comparison to previous studies using similar approaches (see Methods).

We found that a median of 10.23% (range, 1.17–19.65%; mean of 15.03%) of the genome was differentially altered between matched PTs and PDXs across cohorts (Fig. 1a and Supplementary Data 1). Considering all PDX models from all cohorts together, a median of 10.95% (range 0–73.26%; mean of 15.56%) was differentially altered. Overall, over 25% of the genome was differentially affected by CN alterations in about a quarter (24.1%) of the matched PT-PDX samples (Fig. 1b and Supplementary Data 1). Many of the discordances were due to chromosome-arm aberrations that were present in only one of the paired samples: a median of 2 arm-level CN changes was differentially called between PTs and PDXs across cohorts (range, 0 to 4; mean of 3.40; Fig. 1c). The ichorCNA results showed similar discordance values with a median of 7.71% discordance (range 0.33–51.48%; mean of 15.41%; Supplementary Fig. 4a and Supplementary Data 2), 2 arm-level CN changes (range 0–10; mean 3.42; Supplementary Fig. 4b), and over 25% of the genome differentially affected by CN alterations in 21.4% of the matched samples (Supplementary Fig. 4c). Slightly higher discordance values were observed when the analysis was repeated without excluding low-purity samples (Supplementary Fig. 5). Therefore, although high overall correlations were observed between CN profiles of matched PTs and PDXs, such correlation values mask considerable differences between matched samples. Notable examples are shown in Fig. 1d and Supplementary Fig. 4d. Of note, the instability of the PDX models was positively correlated with the degree of CN changes observed in the PTs (Pearson’s *r* = 0.263, *p* = 1.3×10^−4^; Fig. 1e), in line with the strong association between CN heterogeneity and genomic/chromosomal instability, previously demonstrated in both clinical samples and cancer models^2,4,21^.

**Fig. 1:**
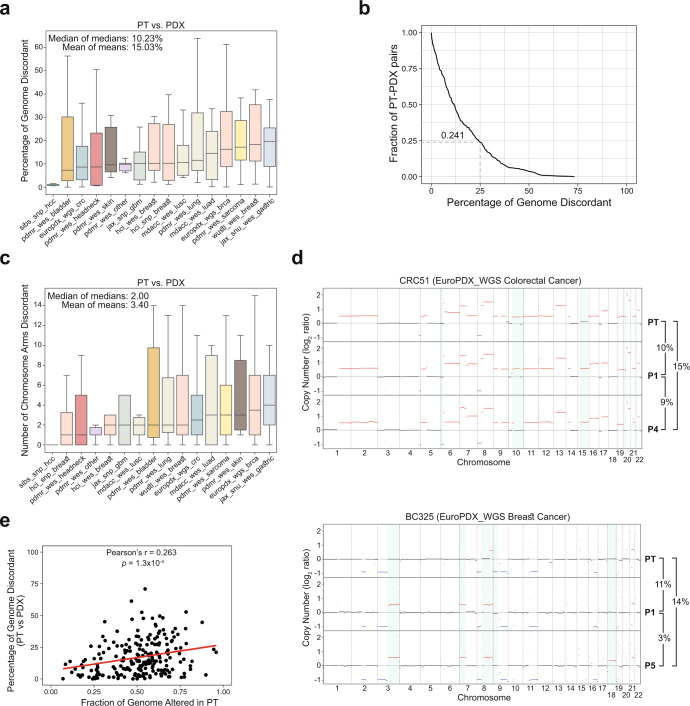
Thresholds-based comparison of the copy number landscapes of PTs and PDXs. **a** A cross-cohort comparison of the percent of the genome that is discordant between matched PT-PDX samples. In the median cohort, a median of 10.23% of the genome is altered between PTs and PDXs. Bar, median; colored rectangle, 25th to 75th percentile; whiskers, Q1 – 1.5*IQR to Q3 + 1.5*IQR; outliers were excluded from the plot. **b** A reverse estimator of cumulative distribution function (1 - eCDF) plot showing the fraction of PT-PDX pairs in which over a given percentage of the genome is discordant. Over 25% of the genome was discordant in 24.1% of the matched PT-PDX samples. **c** A cross-cohort comparison of the number of chromosome arms that are discordant between matched PT-PDX samples. A median of 2 chromosome arms are altered between PTs and PDXs across cohorts. Bar, median; colored rectangle, 25th to 75th percentile; whiskers, Q1 – 1.5*IQR to Q3 + 1.5*IQR; outliers were excluded from the plot. **d** Examples of CN differences between matched PT, an earlier-passage (P1) PDX, and later-passage (P4, P5) PDX samples from the EuroPDX_WGS colorectal and breast cancer cohorts. Red, CN gain; blue, CN loss. Prominent differences are highlighted with a light blue background. The fraction of the genome that is altered between samples is shown to the right of the plot. **e** A scatter plot presenting the correlation between the fraction of the genome affected by CN in the PT and the discordance between that PT and its derived PDX. In general, the more affected by CN a tumor is, the more divergent its PDX is (Pearson’s r=0.26; *p*=1.3 × 10^-4^).

These findings demonstrate that the different magnitudes of PT-PDX discordance reported by previous studies are not due to the use of different platforms for CN calling, but rather due to different definitions of ‘concordance’ and ‘discordance’. To further assess this issue, we analyzed the RNA-based CN calls from the Woo dataset, which included a cohort of hepatocarcinomas and a cohort of gastric tumors. The discordance between the PTs and PDXs in these cohorts was highly similar to that in the HCC and gastric cohorts assessed by SNP and WES, respectively, both in a thresholds-based analysis and in an ichorCNA analysis (Supplementary Fig. 6). Interestingly, both DNA- and RNA-based analyses placed the HCC cohort as the least discordant cohort and the gastric cohort as the second most discordant cohort. These results further confirm that RNA data can be used to assess CN evolution of PDXs, and that the disagreement between previous studies did not stem from the platforms used for CN calling.

A key question is whether genomic evolution with successive passaging leads to PDX diversification away from the genomic structure of primary tumors. Using our measures for CN discordance, we find that the CN discordance between matched pairs of PDXs significantly increases with passaging. In other words, the higher the passage difference, the more discordant matched samples are **(**Fig. 2a and Supplementary Fig. 4e). Notably, the BCM breast cancer cohort is composed of pairs of PDXs 17 to 21 passages apart, the highest passage differences in the entire Woo dataset; by our analysis, this cohort also has the highest median discordance between paired PDXs of any cohort (median, 10.79%; range, 5.61–36.60%; mean, 13.3%). Importantly, however, large differences can also accumulate within few passages, as seen in the EuroPDX colorectal cancer PDX cohort. In both cases, many of the genomic differences involve aneuploidy (i.e., chromosome-arm or whole-chromosome alterations; Fig. 2b,c, Supplementary Fig. 7a and Supplementary Data 1 and 2). Moreover, in cohorts that include ‘trios’ of a PT and a PDX model evaluated at two different passages, the discordance between PTs and later-passage PDXs was significantly higher than that between PTs and earlier-passage PDXs (*p* = 0.0003 and *p* = 0.0007, one-sided Wilcoxon signed-rank test; for the breast and colon cohorts, respectively; Fig. 2d and Supplementary Fig. 7b).

**Fig. 2:**
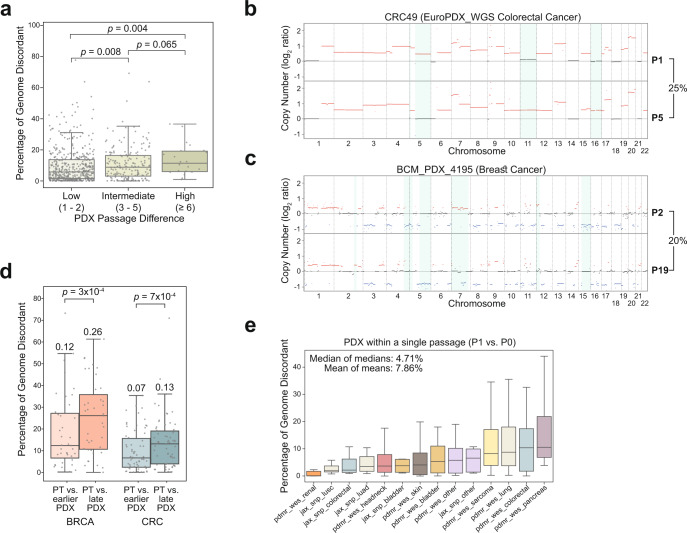
Thresholds-based comparison of the copy number landscapes of PDXs at different passages. **a** A comparison of the percent of the genome that is discordant between matched samples of PDXs with a low (1–2), intermediate (3–5) or high (>=6) passage difference between them. The discordance increases with passage difference. *P* values obtained by Mann-Whitney U test. Circles, individual pairs. **b**, **c** Examples of the CN differences between matched early passage and late passage PDX samples from the EuroPDX_WGS colorectal and breast cancer cohorts. Red, CN gain; blue, CN loss. Prominent differences are highlighted with a light blue background. The fraction of the genome that is altered between samples is shown to the right of the plot. **d** A comparison of the percent of the genome that is discordant between PTs vs. earlier-passage PDXs and PTs vs. later-passage PDXs, in breast and colorectal cancer cohorts that included matched ‘trios’ of PT and PDXs from two passages. P-values obtained by a one-sided Wilcoxon signed-rank test. Bar, median; colored rectangle, 25th to 75th percentile; whiskers, Q1 – 1.5*IQR to Q3 + 1.5*IQR; outliers were excluded from the plot Circles, individual pairs. **e** A cross-cohort comparison of the percent of the genome that is discordant between matched samples of PDXs at passage 0 and passage 1. A median of 4.71% of the genome is altered between P0 and P1 across cohorts. Bar, median; colored rectangle, 25th to 75th percentile; whiskers, Q1 – 1.5*IQR to Q3 + 1.5*IQR; outliers were excluded from the plot.

We note that our pancancer analysis is not controlled for passage number, which can vary considerably between PDX cohorts (Supplementary Data 1). To assess the extent of genetic changes occurring within a single passage, we therefore analyzed the genomic differences between P1 and P0 PDXs. Across all cohorts, a median of 4.71% (range, 0.093–10.50%; mean, 7.86%) of the genome was altered between P0 and P1 of the PDX models (Fig. 2e and Supplementary Fig. 8). This is also consistent with our previous observation that genomic evolution is particularly extensive within the first few PDX passages^4^. These findings provide evidence for a gradual genetic diversification of PDXs throughout their engraftment and passaging.

In our previous study, we did not identify specific CNAs that were selected for in the PDX models^4^ due to a limited number of samples when controlling for tumor type. We confirmed that the sample sizes in the Woo dataset are not statistically powered (Type II Error <80%) to detect a 10% CNA prevalence decrease for nearly all (96%) of the TCGA-recurrent aneuploidies (Supplementary Fig. 9). Therefore, the ability to identify specific recurrent mouse-induced CNAs and the presence or absence of selection will require much larger cohorts. We nonetheless analyzed the genomic regions affected by CN, using two approaches. First, we performed a gene-level prevalence analysis in each tumor type. The prevalence of gene-level CN discordance was computed for all the genes that reside within discordant regions (between PTs and PDXs, and between early-passage and late-passaged PDXs) and gene set enrichment analysis (ssGSEA) was performed. We did not observe any consistently enriched or depleted pathways shared by all cohorts. However, significant enrichments were observed in specific tumor types (Supplementary Fig. 10a,b and Supplementary Data 3). For example, in several tumor types the CNAs that differed between early and later PDX passages were enriched for genes associated with immune-response-related pathways (Supplementary Fig. 10b and Supplementary Data 3). Next, we curated a list of cancer genes from the COSMIC Cancer Gene Census database^22^ and examined the prevalence of their CNA discordance in each tumor type (comparing PTs vs. PDXs, and early-passage vs. late-passage PDXs). Overall, 22.7% of the cancer genes resided in regions that were discordant between PTs and PDXs in >20% of the tumors (Supplementary Fig. 10c), and ~42.3% of the genes resided in CNAs that differed between early and later PDXs in >10% of the tumors (Supplementary Fig. 10d, Supplementary Data 4). The number of genes within commonly discordant CNAs varied considerably across cohorts: whereas in some cohorts (e.g., pdmr_wes_renal) no cancer genes recurrently differed between PTs and PDXs, in other cohorts (e.g., europdx_wgs_brca) a large fraction of the cancer genes did recurrently differ (Supplementary Fig. 11 and Supplementary Data 4). We identified 90 genes that were commonly discordant (in >25% of the PT-PDX pairs) in ≥ 5 tumor cohorts (Supplementary Fig. 12 and Supplementary Data 4), including common oncogenes such as BRAF and BCL2. While strictly correlative, these findings suggest that the CNAs arising during model evolution have the potential to alter important biological pathways.

Importantly, clonal selection may not necessarily involve recurrent events. When the same PT is transplanted into different mice, the ‘sibling’ PDX models tend to acquire the same CNAs, indicating positive selection^4^. Moreover, the rate of CN evolution in PDXs is strongly correlated with the intra-tumor heterogeneity of the primary tumor types^4^, which is also supported by observations that the CN differences between PTs and PDXs are similar to the CN differences across multi-region tumor samples^5^. We note that the observation that PDX genomic evolution is largely driven by spatial heterogeneity by no means diminishes its potential consequences; PDX evolution due to spatial heterogeneity could still lead to considerable differences between PTs and their derived PDXs, which could potentially reduce the utility of such divergent models. Importantly, the finding that the later passage PDXs are significantly less similar to their matched PTs than the earlier passage PDXs cannot be explained by the spatial heterogeneity within the PT.

Our analysis underestimates the genomic diversification of PDX models. First, high-level amplification or deep deletions are not considered as changes compared to single copy gains or losses of the same regions. Second, our analysis is blind to copy-neutral LOH events. Third, whole-genome duplication events, single-nucleotide alterations (SNVs) and structural alterations (SVs) are not considered either. Therefore, our results provide a conservative estimate of the minimum fraction of the genome that is actually altered throughout PDX engraftment and passaging. Interestingly, our results are in full agreement with a recent study that analyzed the evolution of 536 PDX models, focusing on SNVs, SVs and gene-level LOH events, which reported ~10% discordance in key driver mutations between PTs and their matched PDXs^6^. Importantly, the rate of PDX evolution that we observe is also similar to that seen in cancer cell lines, and is significantly associated with the stability of the tumor of origin (Fig. 1e; reviewed in^19^). Therefore, PDXs are subjected to the same evolutionary processes that act on the native tumor and on alternative tumor models, but the specific selection pressures would be context-dependent. We note that some PDX models may indeed have highly stable CN profiles, but our data suggest that it is important to validate this in order to correctly interpret experimental results obtained using PDX models.

In summary, our analysis of CN profiles from PDXs reconciles the apparent contradictions previously reported, constituting a DNA-based validation of PDX genomic evolution. Importantly, the disagreement between previous studies is not a matter of the platform used for CN calling, but was largely driven by different methods of data analysis and interpretation (including the exact CN evaluation methods). Ultimately, the data from the three recent large-scale studies (Ben-David et al., Woo et al., and Sun et al.) are highly consistent, documenting on average 10–20% CN discordance between PTs and PDXs, and revealing ongoing diversification of CN landscapes throughout model propagation. The source of this diversification is probably a combination of subclonal dynamics and ongoing genomic instability (reviewed in^19^). Important questions remain, however, with regard to the functional consequences of this phenomenon: Is it alarming or negligible that >10% of the genome is different between PTs and PDXs, that these CN alterations often include chromosome-arm gains and losses, and that the genomic similarity of PDXs to their PT of origin decreases over time? How do such changes affect the phenotypic stability of the models, and their ability to serve as accurate ‘tumor avatars’? Addressing these questions directly and systematically is important for optimizing the use of these unique and valuable cancer models.

## Methods

### PDX dataset and preprocessing

Segments of log_2_(CN ratio) values across the genome for both PT and PDX samples were acquired directly from the supplementary tables in the Woo et al.^5^ study. The log_2_(CN ratio) values for segmented data were generated for various platforms; details are provided in the Woo, et al study. In short, the log_2_ ratio value for each data point i was defined as

1 $$\log _2\left( {ratio} \right) = \log \{ (\frac{{\theta _i}}{{\theta _{ref}}}\} ) = \bar \theta _i$$

where $\theta _i$ is the normalized intensity or read count and $\theta _{ref}$ is the normalized (average) value for normal or reference sample(s), depending on the platform. Segmentation algorithms applied to these data points by Woo et al. resulted in segments used as input in this current study and for each segment ${{{\mathrm{s}}}}$, the

2 $$\log _2\left( {CN\,ratio} \right) = \mathop {\sum}\limits_{i \in s} {\bar \theta _i} .$$

Samples whose CN profiles had been estimated from SNP arrays, whole-exome sequencing data, and low-pass whole-genome sequencing data were included, while samples whose profiles had been estimated from RNA-sequencing and gene expression microarray data were excluded, to avoid potential issues with expression-based CN inference. The final dataset consisted of 1,429 samples across 33 cohorts containing at least one pair of matched samples.

In the Woo et al. study, the authors divided the genome into equal-sized 100 kB bins, assigned the log_2_(CN ratio) of the overlapping segment at each bin, and compared the log_2_(CN ratio) values of the bins between pairs of samples using a Pearson correlation (without copy number determination).

For this current study, the log_2_(CN ratio) segments for each sample were binned into 1 Mb windows across the genome. Bins that overlapped more than one segment were assigned the value of the overlapping segment with greatest absolute value.

### Sample pairing scheme

For analyzing PT vs. PDX and PDX vs. PDX discordance across different cohorts, each PT sample was compared to each of its available PDXs. Each PDX sample was compared to every other PDX available from the same original PT, provided that the PDX samples were of different passage numbers. Cohorts with <5 PT-PDX or PDX-PDX pairs were excluded from the respective analyses.

For analyzing PDX vs. PDX discordance by passage difference, PDX samples were paired with later-passage PDX samples in the same PDX model with the minimum possible passage difference.

For analyzing trios from the EuroPDX WGS cohorts, each model with a PT and two PDX samples of different passage numbers was evaluated by comparing the PT to both PDXs in order to calculate the discordance between PT and the earlier-passage PDX and that between the PT and the later-passage PDX.

### Computing CNA discordance between paired samples using thresholds

CN gains and losses were defined as log_2_(CN ratio) >=0.3 or log_2_(CN ratio) <=-0.3, respectively. For a 1 Mb bin to be discordant between two samples, one sample must uniquely have a CN gain or loss, and additional criteria specifying the difference in log_2_(CN ratio) required between the two samples must be satisfied, to exclude borderline cases. In full, if two samples have log_2_(CN ratio) values A and B, the samples are discordant if at least one of the following conditions are met:

3 $$\begin{array}{l}({{{\mathrm{A}}}} > = 0.3\;\& \;{{{\mathrm{B}}}} < 0.3)\;\& \;(\left( {{{{\mathrm{A}}}}--{{{\mathrm{B}}}}} \right) > = 0.3|({{{\mathrm{B}}}} < 0.1))\\ ({{{\mathrm{A}}}} < = - 0.3\;\& \;{{{\mathrm{B}}}} > - 0.3)\;\& \;(\left( {{{{\mathrm{A}}}}--{{{\mathrm{B}}}}} \right) < = - 0.3|({{{\mathrm{B}}}} > - 0.1))\\ ({{{\mathrm{B}}}} > = 0.3\,\& \,{{{\mathrm{A}}}} < 0.3)\,\& \,(\left( {{{{\mathrm{B}}}}--{{{\mathrm{A}}}}} \right) > = 0.3|({{{\mathrm{A}}}} < 0.1))\\ ({{{\mathrm{B}}}} < = - 0.3\,\& \,{{{\mathrm{A}}}} > - 0.3)\,\& \,\left( {\left( {{{{\mathrm{B}}}}--{{{\mathrm{A}}}}} \right) < = - 0.3|\left( {{{{\mathrm{A}}}} > - 0} \right.} \right..\end{array}$$

The fraction of 1 Mb bins discordant across the genome between two samples is defined as the number of discordant bins divided by the number of bins across the genome for which both samples have log_2_(CN ratio) values.

For a chromosome arm to be discordant between two samples, >=75% of the 1 Mb bins in the chromosome arm must be discordant using the above thresholds, and these discordances must always be due to one sample having the greater log_2_(CN ratio) and the other sample having the lesser log_2_(CN ratio).

To exclude highly-discordant cases that may be due to tumor purity issues, a sample was assumed to lack sufficient tumor fraction to analyze if <5% of the 1 Mb bins in its genome with log_2_(CN ratio) values had absolute values of >=0.3. 100 samples were removed from analysis due to this criterion.

### Copy number analysis using ichorCNA

For each sample, the log_2_(CN ratio) values at each 1 Mb bin were passed in as input to a modified version of the Hidden Markov Model-based algorithm ichorCNA(11). This version of ichorCNA calls large-scale CNAs and estimates tumor fraction and ploidy from a sample’s median-normalized, GC-corrected log_2_(CN ratio) values across the genome. ichorCNA uses a probabilistic model to predict CNAs and does not use log_2_(CN ratio) thresholds. PT samples were run with normal cell fraction initializations of 0.25, 0.5, and 0.75, and PDX samples were initialized with 0.05 normal cell fraction. Samples were run for starting conditions of both ploidy=2 and ploidy=3. ichorCNA selects a sample’s final solution based on the likelihood scores of the set of solutions produced from the different runs.

If the ploidies of each set of paired samples in a model did not converge to within 0.2 of each other, some of the samples were rerun with their starting ploidies restricted to one value to increase the likelihood of each sample having similar estimated ploidy. The details of this approach depended on the composition of the model: If a model was composed of one PT and one PDX, the PT sample was rerun with the closest possible starting ploidy to the PDX’s estimated ploidy. If a model was composed of two PDXs, the sample with lower tumor fraction was rerun with the closest possible starting ploidy to its pair’s estimated ploidy, and if these samples had equal tumor fraction, the sample with higher ploidy was rerun. Finally, if a model was composed of more than two samples, the starting ploidy was restricted to the most common rounded estimated ploidy. In the case of multiple equally common rounded estimated ploidies, the new starting ploidy was picked randomly from those ploidies.

### Computing CNA discordance between paired samples from ichorCNA results

CNA profiles across the genome were transformed using the equation:

4 $${{{\mathrm{Ploidy}}}}\,{{{\mathrm{adjusted}}}}\,{{{\mathrm{CN}}}} = {{{\mathrm{CN}}}}\,/\,{{{\mathrm{ploidy}}}} \ast {{{\mathrm{2}}}}$$

Rounded, ploidy-adjusted CN of >2 was defined as a CN gain, and <2 as a loss. For a 1 Mb bin to be discordant between two samples, one sample must uniquely have a CN gain or loss, and the unrounded ploidy-adjusted CNs must also differ between the two samples by > = 0.5.

ichorCNA calls some 1 Mb bins as subclonal CN gains/losses. Subclonal CN calls with >90% estimated cellular prevalence were treated as clonal gains/losses, and subclonal CN calls with <10% cellular prevalence were treated as neutral CN. These calls were then subjected to the same ploidy-adjustment as other clonal calls.

Subclonal copy number calls with intermediate cellular prevalence (between 10 and 90%, inclusive) were not considered for computing discordance.

Chromosome arm level discordance between two samples was again defined as > = 75% discordance of the 1 Mb bins in an arm, with one sample always having the greater CN and the other sample having the lesser CN.

Samples were excluded from analysis if ichorCNA estimated that the sample contained <5% tumor fraction and/or if <5% of the bins were clonally altered from neutral CN after ploidy-adjustment and rounding. 52 samples were excluded based on these criteria.

### Advantages of our copy-number discordance calculations

1. **Importance of direct inference of copy number events**. Our discordance estimation is based directly on the inferred copy number. To obtain copy number, we applied two approaches: (1) thresholding criteria on the log_2_(ratio) values; and (2) ichorCNA, which accounted for data variability, tumor purity, ploidy, and potential mouse DNA contamination. For both approaches, we computed the percentage of the genome that showed discordance based on copy number changes. By contrast, Woo et al. compared pairs of samples using the Pearson correlation between normalized log2 ratio data, which are typically the input data into copy number algorithms.
2. **Importance of direct copy number discordance calculation**. The pitfalls of the Pearson correlation approach include the masking of true CNA discordance from smaller segments and more extreme copy number values. Furthermore, the degree of concordance can be arbitrary because interpretation of the correlation coefficient is challenging. By contrast, our approach evaluates the actual CNA events that lead to breaks in the genome, leveraging accepted and conventional ways to analyze CNA. Furthermore, we use the fraction-of-the-genome-discordant metric, which is widely used for measuring genomic and chromosomal instability. The discordance results between ichorCNA and the thresholds-based strategy exhibit high level of agreement (Supplementary Fig. 3), demonstrating the consistency of our results despite using two entirely different approaches for copy number calling.
3. **Importance of direct comparisons with related studies**. As recent studies (Sun et al. and Ben-David et al.) also used the discordance metric for estimating the magnitude of PDX evolution, this approach is more appropriate for direct comparisons between datasets and studies.

### Gene discordance analysis

For each 1 Mb bin, the proportion of discordance across pairs within a cohort was computed. All protein-coding genes were assigned a proportion of discordance based on the overlapping bin with the gene boundaries. If more than one bin overlapped the gene boundaries, the average proportion across the overlapping bins was used. Cancer genes were determined by the 704 Tier 1 and 2 Cancer Gene Census (https://cancer.sanger.ac.uk/cosmic/census?tier=all) from the COSMIC database^22^.

Gene set enrichment was performed using over-representation analysis for MSigDB Hallmarks (H). For each cohort, the top 2.5% of most discordant genes with a proportion >0.25 were selected from the full set of protein-coding genes. Cohorts with no genes meeting the criteria of >0.25 proportion were excluded. A hypergeometric test was performed for the selected genes and for each Hallmark. The enrichment score (ES) for hallmark gene set ${{{\mathrm{s}}}}$ and cohort $c$ was defined as

5 $${{{\mathrm{ES}}}}_{{{{\mathrm{s}}}},{{{\mathrm{c}}}}} = \log \left( {\frac{{x_{s,c}}}{{N_c}}/\frac{{g_s}}{G}} \right)$$

where ${{{\mathrm{N}}}}_{{{\mathrm{c}}}}$ is the number of selected genes for cohort $c$; $x_{s,c}$ is the number of genes from the $N_c$ selected genes that are in hallmark set ${{{\mathrm{s}}}}$ and cohort $c$; $g_s$ is the total number of genes in hallmark ${{{\mathrm{s}}}}$; and ${{{\mathrm{G}}}}$ is the total of number of genes from all hallmark sets combined. The p-values were adjusted for multiple hypothesis testing using Benjamini-Hochberg method.

### Statistical analysis

The statistical significance of the difference in genomic discordance between PTs vs. early-passage PDXs and PTs vs. later-passage PDXs was determined by one-sided Wilcoxon signed-rank test. The statistical significance of the difference in genomic discordance between matched PDX pairs with low (1–2), intermediate (3–5) or high (≥6) passage number difference was determined by Mann-Whitney U test. Statistical analyses were performed using the SciPy Python library. Plotting was performed using the Pandas and Seaborn Python libraries.

### Power analysis for PDX selection against recurrent aneuploidies analysis

The prevalence of specific aneuploidies in patient tumors for a given tumor type was calculated from the aneuploidy calls of 10,522 cancer genomes from The Cancer Genome Atlas (TCGA) as presented in Taylor et al.^23^. Arm-level alterations with prevalence >0.25 were defined as recurrent. We determined the minimum sample size n needed to detect a 10% absolute decrease in the prevalence of a recurrent arm for a specific tumor type with sufficient power of 80%, using a one-sided one-sample proportion test.

6 $${{{\mathrm{n}}}} = p_0\left( {1 - p_0} \right)\left( {\frac{{z_{1 - \alpha } + z_{1 - \beta \sqrt {\frac{{p\left( {1 - p} \right)}}{{p_0\left( {1 - p_0} \right)}}} }}}{{p - p_0}}} \right)^2$$

The reference p_0_ represents the expected prevalence of the recurrent arm in the TCGA patient tumors for a specific tumor type, while *p* represents the scenario for a 10% prevalence decrease; *p* = *p*_0_ – 0.1. z is the inverse of the cumulative distribution function with Type I error α = 0.05, Type II error β = 0.2, and power 1-β = 0.8. For each recurrent arm of a given tumor type, we compared the powered sample size n to the number of PT-PDX comparisons available in our dataset for the given tumor type.

### Reporting summary

Further information on research design is available in the Nature Research Reporting Summary linked to this article.

## Supplementary information

Supplementary Information

Supplementary Table 1

Supplementary Table 2

Supplementary Table 3

Supplementary Table 4

REPORTING SUMMARY

## Data Availability

All datasets used in this analysis are publicly available in Supplementary Data 1 in the Woo et al study^5^ (Gene Expression Omnibus accession umbers GSE90653, GSE3526 and GSE33006 and ArrayExpression accession number E-MTAB-1503-3).

## Code availability

The codes used to analyze the data are publicly accessible at https://github.com/GavinHaLab/pdx_evolution_study.
