# Publisher Correction: DNA-based copy number analysis confirms genomic evolution of PDX models

**DOI:** 10.1038/s41698-022-00293-5

**Published:** 2022-07-12

**Authors:** Anna C. H. Hoge, Michal Getz, Anat Zimmer, Minjeong Ko, Linoy Raz, Rameen Beroukhim, Todd R. Golub, Gavin Ha, Uri Ben-David

**Affiliations:** 1 Public Health Sciences Division, Fred Hutchinson Cancer Research Center, Seattle, WA USA; 2 Department of Human Molecular Genetics and Biochemistry, Faculty of Medicine, Tel Aviv University, Tel Aviv Israel; 3 Broad Institute of Harvard and MIT, Cambridge, MA USA; 4 Dana-Farber Cancer Institute, Boston, MA USA; 5 Harvard Medical School, Boston, MA USA

**Keywords:** Cancer genomics, Cancer models

Correction to: *npj Precision Oncology* 10.1038/s41698-022-00268-6, published online 28 April 2022

In the original version of this article, the given and family names of Uri Ben-David were incorrectly structured. The original article has been corrected.

