# Supplementary material for: DNA-based copy number analysis confirms genomic evolution of PDX models
Source: NPJ Precis Oncol. 2022 Apr 28;6:30. doi: 10.1038/s41698-022-00268-6 (PMC9050710; doi:10.1038/s41698-022-00268-6)
Supplement: Supplementary file 1 — Supplementary Information [file 41698_2022_268_MOESM1_ESM.pdf]

a

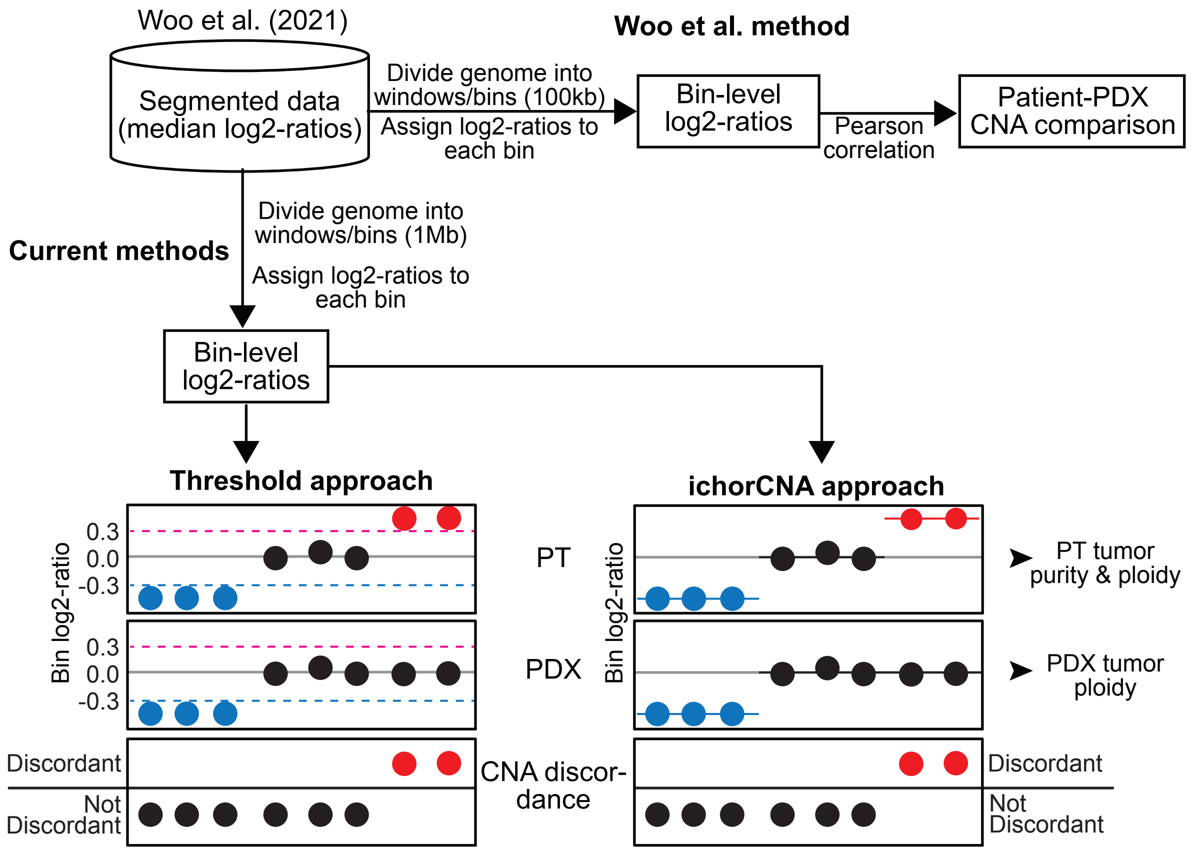

Supplementary Figure 1

b

Threshold approach

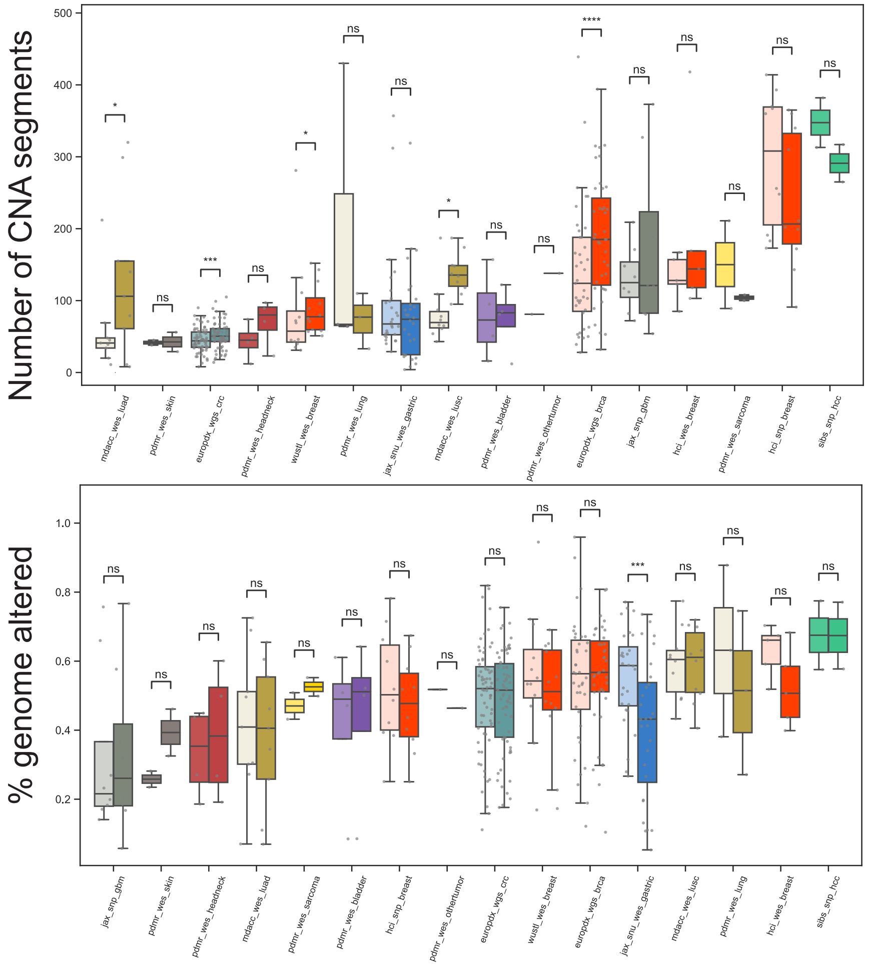

c

ichorCNA approach

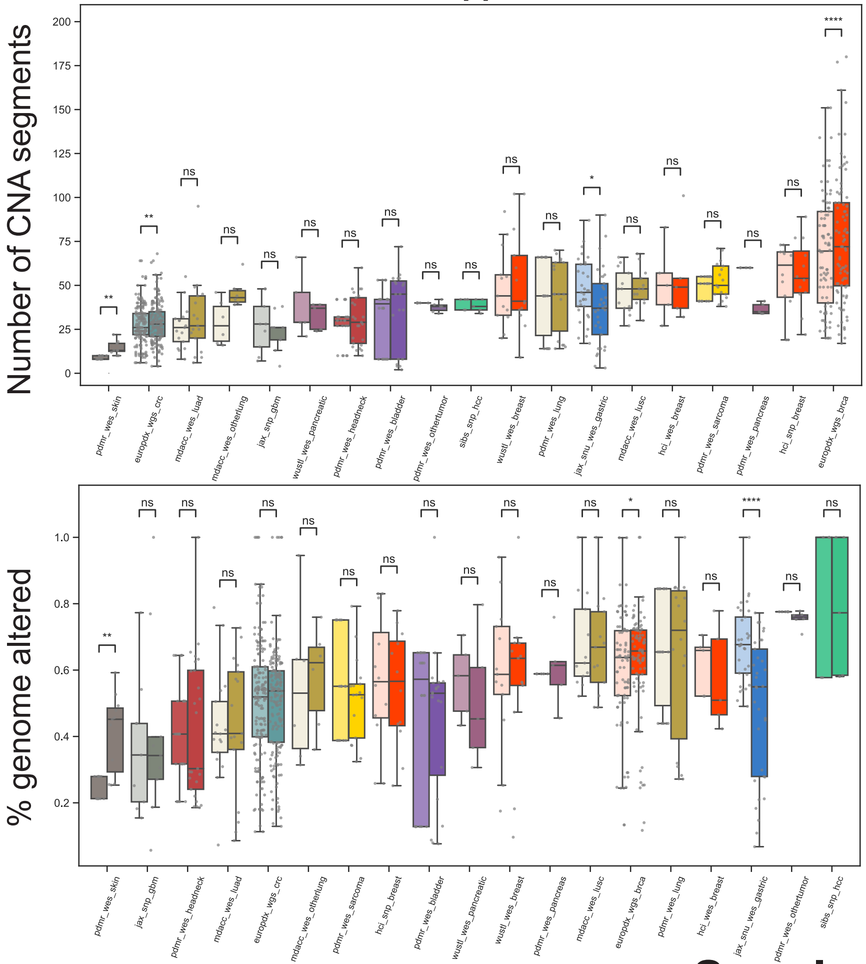

Supplementary Figure 1

Supplementary Figure 1: Similar levels of CN alterations in PTs and PDXs. (**a**) Schematic presentation of the methods of the methods used to infer the degree of discordance between PTs and PDXs in the study by Woo et al. and in the current study. (**b**) A cross-cohort comparison of the number of copy number segments (top) or the fraction of the genome that is affected by CNAs (bottom) between matched PT-PDX samples. The comparison is based on the thresholds-based CN calling (see **Methods**). (**c**) A cross-cohort comparison of the number of copy number segments (top) or the fraction of the genome that is affected by CNAs (bottom) between matched PT-PDX samples. The comparison is based on the ichorCNA-based CN calling (see **Methods**). Bar, median; colored rectangle, 25th to 75th percentile; whiskers, Q1 – 1.5*IQR to Q3 + 1.5*IQR; outliers were excluded from the plot.

a

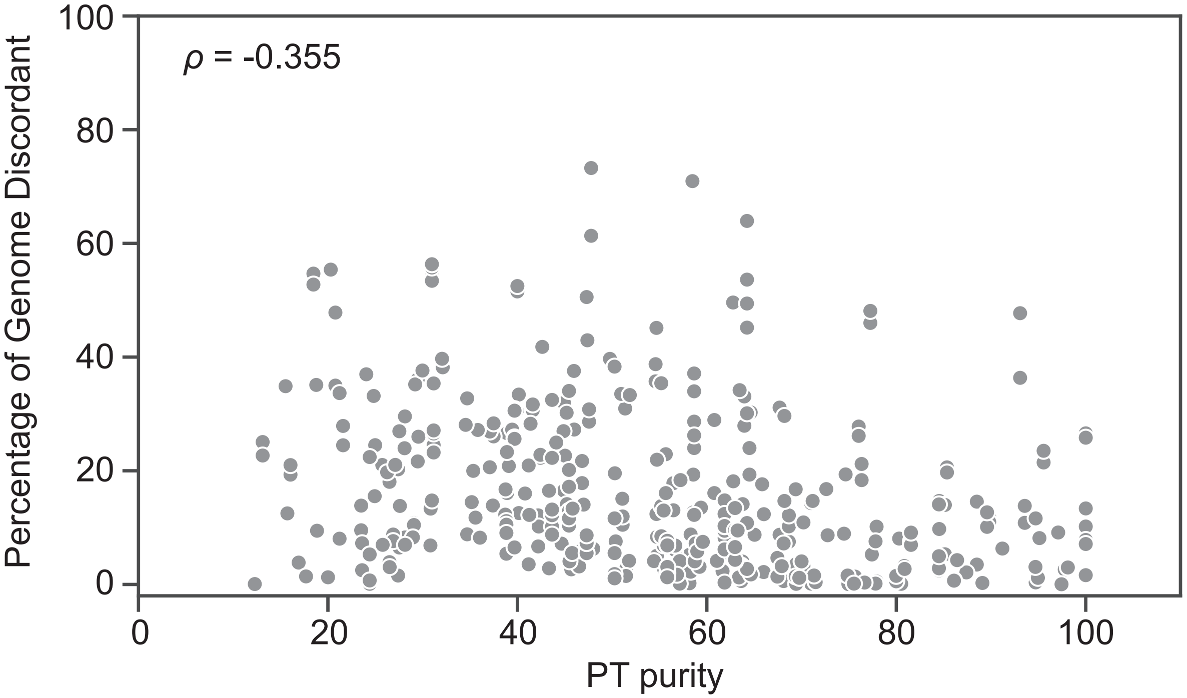

b

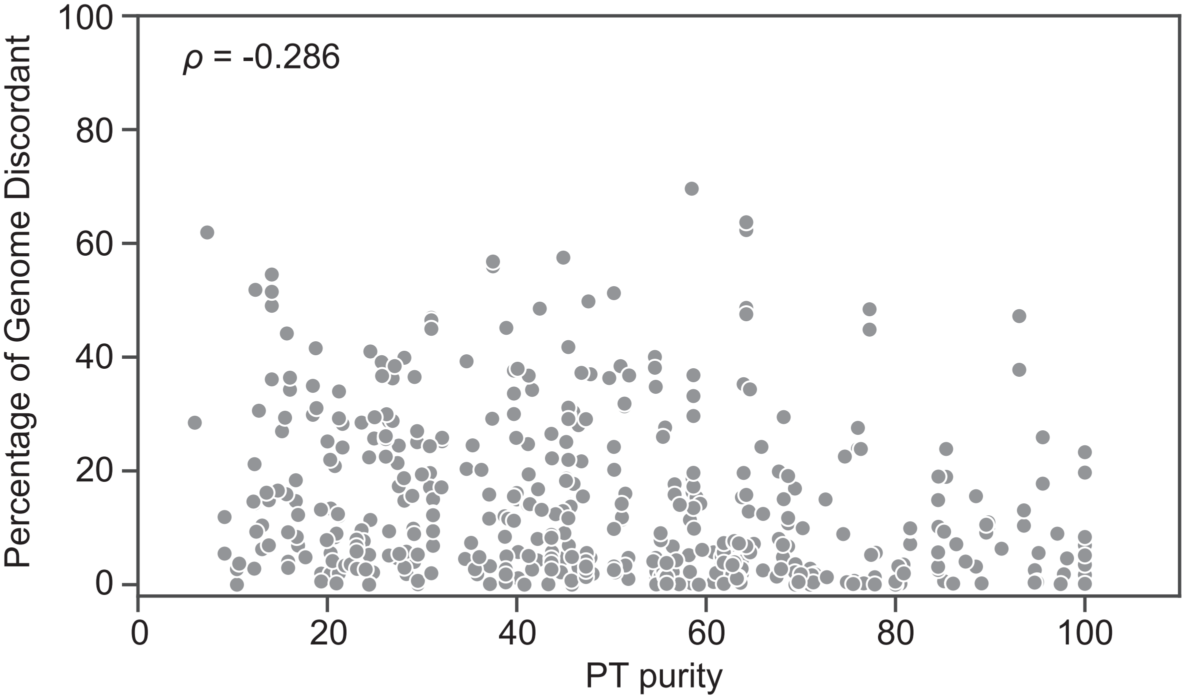

Supplementary Figure 2

Supplementary Figure 2: The contribution of purity to CN discordance. (**a**) The Spearman correlation between the PT purity estimate and the percent of the genome that is discordant between the PT and its matched PDX in the thresholds-based CN analysis. (**b**) The Spearman correlation between the PT purity estimate and the percent of the genome that is discordant between the PT and its matched PDX in the ichorCNA-based CN analysis. Data points correspond to PT-PDX matched samples.

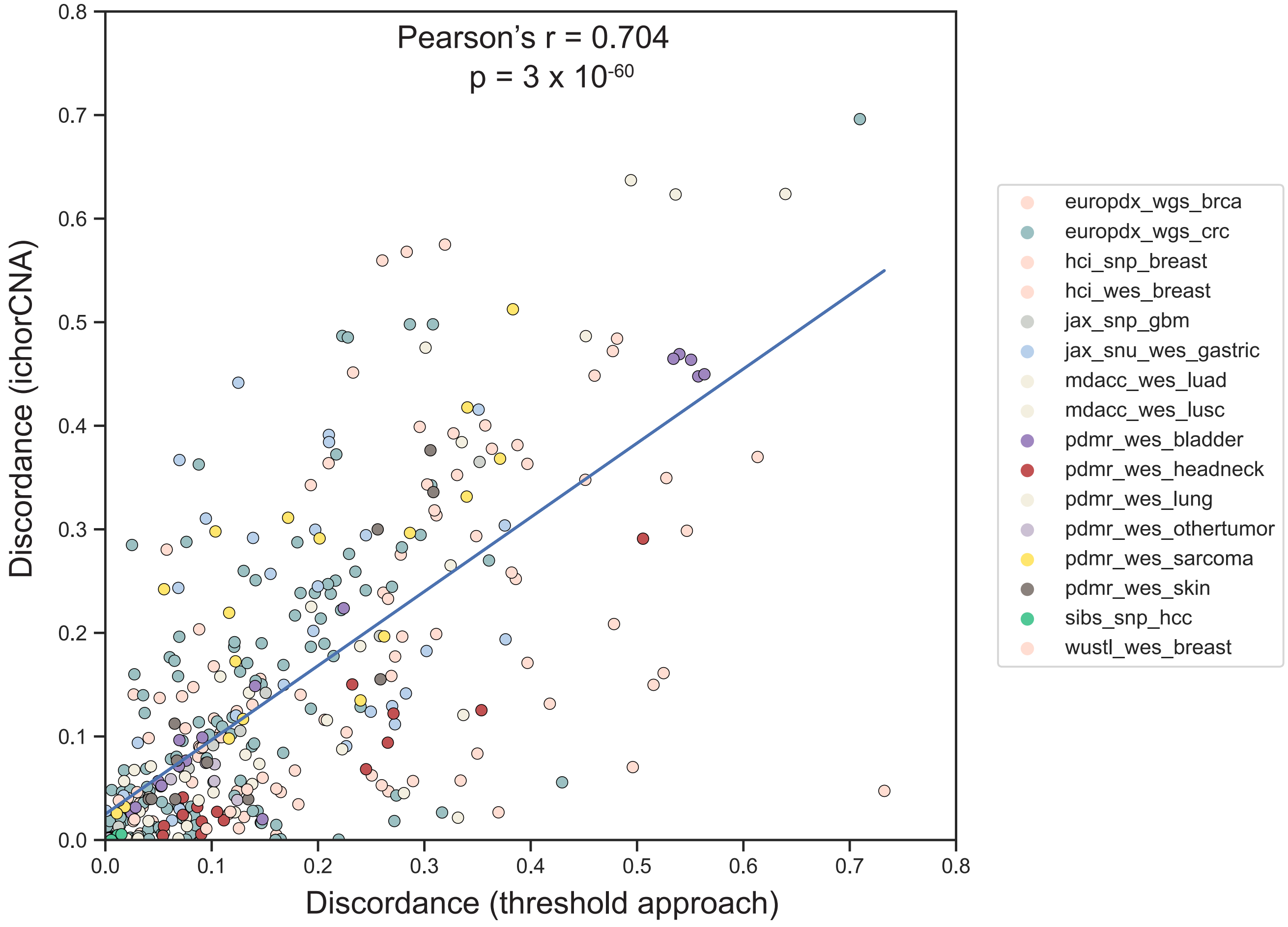

Supplementary Figure 3

Supplementary Figure 3: High concordance between different CNA calling methods. A scatter plot showing the correlation between the PT-PDX discordance values obtained by a thresholds-based analysis (**Fig. 1**; see **Methods**) and an ichorCNA-based analysis (**Supplementary Fig. 4**; see **Methods**).

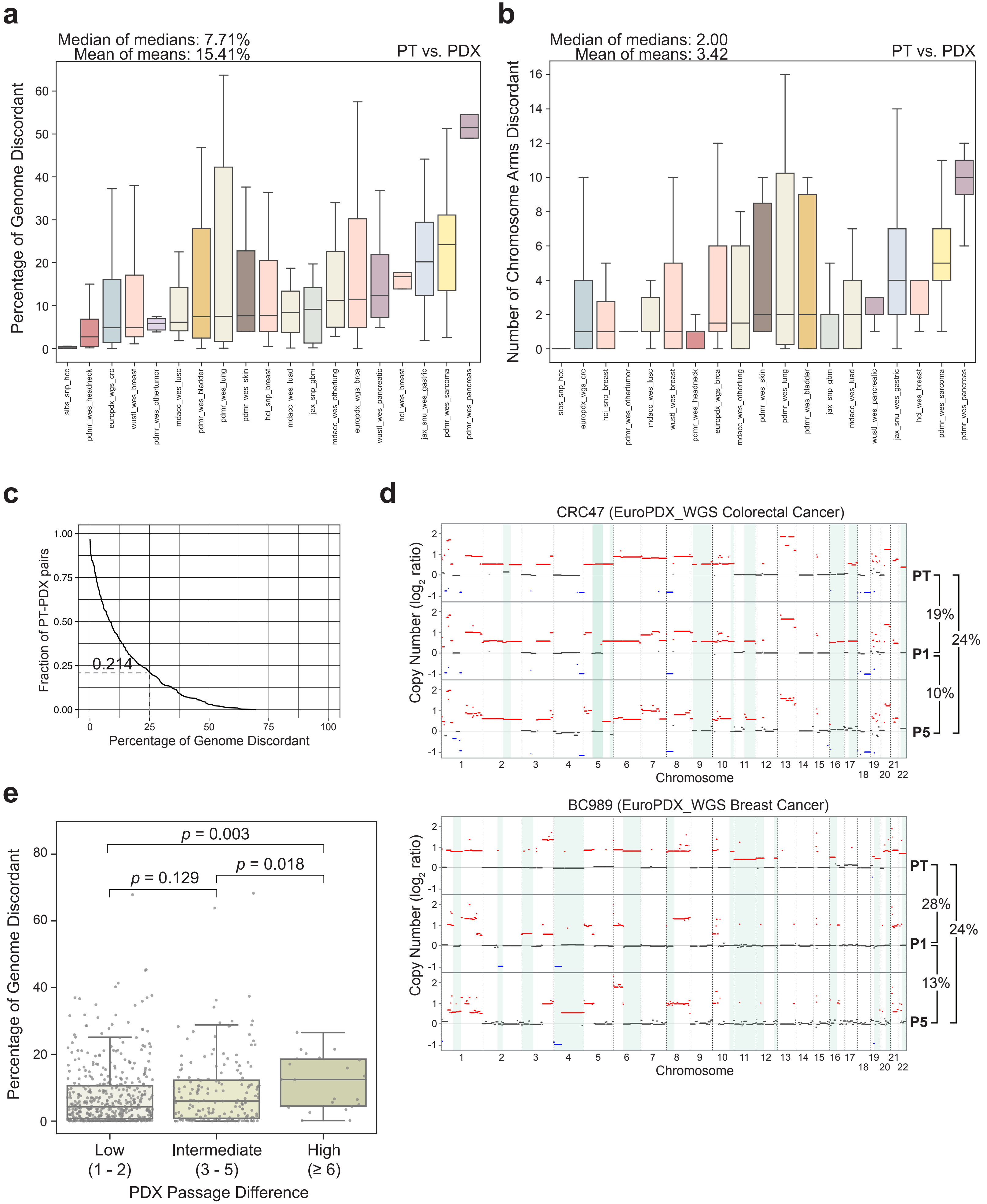

Supplementary Figure 4

Supplementary Figure 4: ichorCNA-based comparison of the copy number landscapes of PTs and PDXs. (**a**) A comparison of the percent of the genome that is discordant between matched PT-PDX samples. In the median cohort, a median of 7.71% of the genome is altered between PTs and PDXs. Bar, median; colored rectangle, 25th to 75th percentile; whiskers, Q1 – 1.5*IQR to Q3 + 1.5*IQR; outliers were excluded from the plot.. (**b**) A comparison of the number of chromosome arms that are discordant between matched PT-PDX samples. In the median cohort, a median of 2 chromosome arms are altered between PTs and PDXs. Bar, median; colored rectangle, 25th to 75th percentile; whiskers, Q1 – 1.5*IQR to Q3 + 1.5*IQR; outliers were excluded from the plot. (**c**) A reverse estimator of cumulative distribution function (1 - eCDF) plot showing the fraction of PT-PDX pairs in which over a given percentage of the genome is discordant. Over 25% of the genome was discordant in 21.4% of the matched PT-PDX samples. (**d**) Examples of ichorCNA results showing the CN differences between matched PT, earlier-passage (P1) PDX and later-passage (P5) PDX samples from the EuroPDX_WGS colorectal and breast cancer cohorts. Red, CN gain; blue, CN loss. Prominent differences are highlighted with a light blue background. The fraction of the genome that is altered between samples is shown to the right of the plot. (**e**) A comparison of the percent of the genome that is discordant between matched samples of PDXs with a low (1-2), intermediate (3-5) or high (≥6) passage difference between them. The discordance increases with larger passage differences. P-values indicate obtained by a Mann-Whitney U test. Circles, individual pairs. Bar, median; colored rectangle, 25th to 75th percentile; whiskers, Q1 – 1.5*IQR to Q3 + 1.5*IQR.

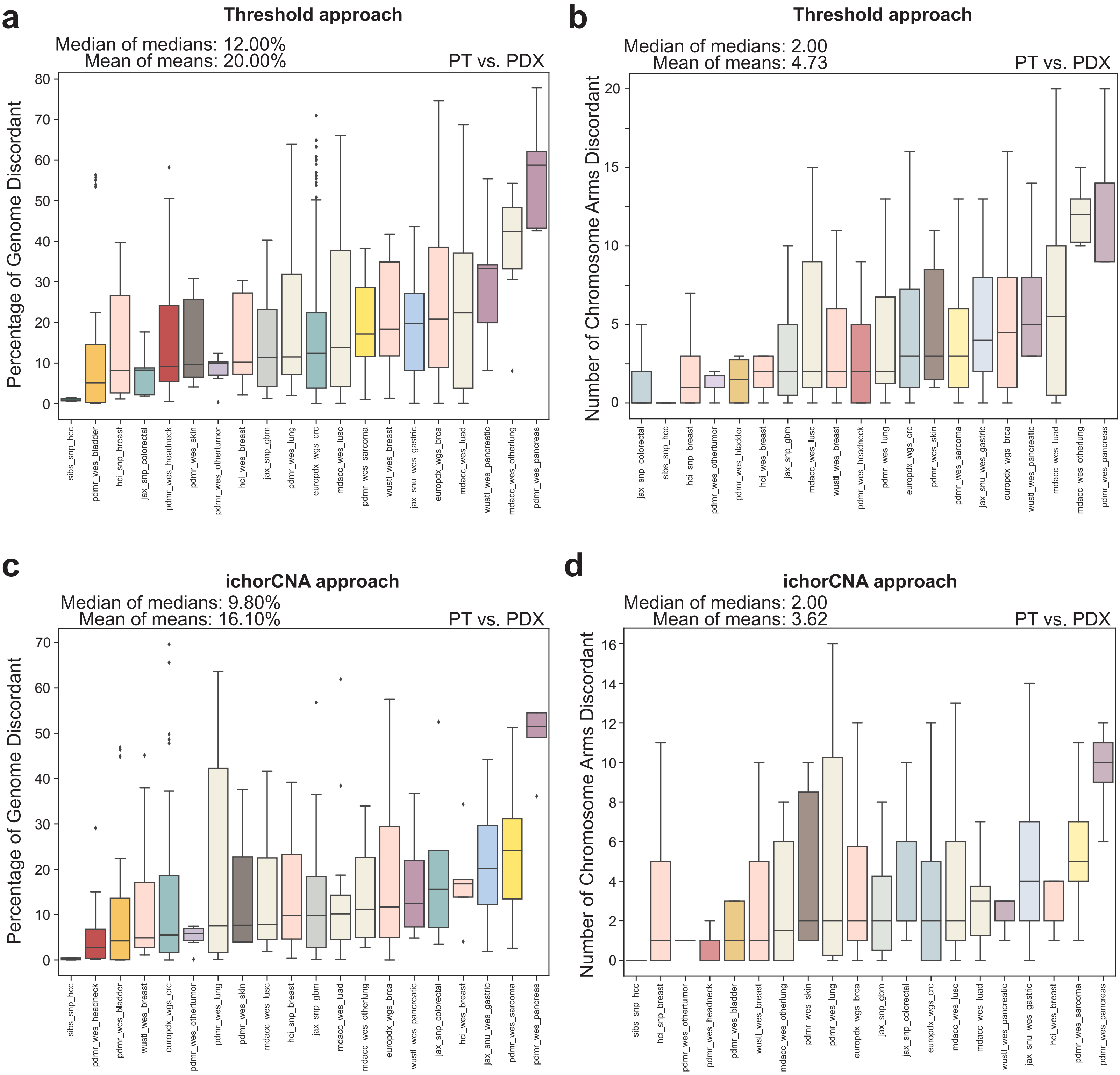

Supplementary Figure 5

Supplementary Figure 5: Discordance analysis with no sample exclusion. (**a**) A thresholds-based comparison of the percent of the genome that is discordant between matched PT-PDX samples. In the median cohort, a median of 12.00% of the genome is altered between PTs and PDXs. (**b**) A thresholds-based comparison of the number of chromosome arms that are discordant between matched PT-PDX samples. A median of 2 chromosome arms are altered between PTs and PDXs across cohorts. (**c**) An ichorCNA-based comparison of the percent of the genome that is discordant between matched PT-PDX samples. In the median cohort, a median of 9.80% of the genome is altered between PTs and PDXs. (**d**) An ichorCNA-based comparison of the number of chromosome arms that are discordant between matched PT-PDX samples. A median of 2 chromosome arms are altered between PTs and PDXs across cohorts. Bar, median; colored rectangle, 25th to 75th percentile; whiskers, Q1 – 1.5*IQR to Q3 + 1.5*IQR; outliers were excluded from the plot.

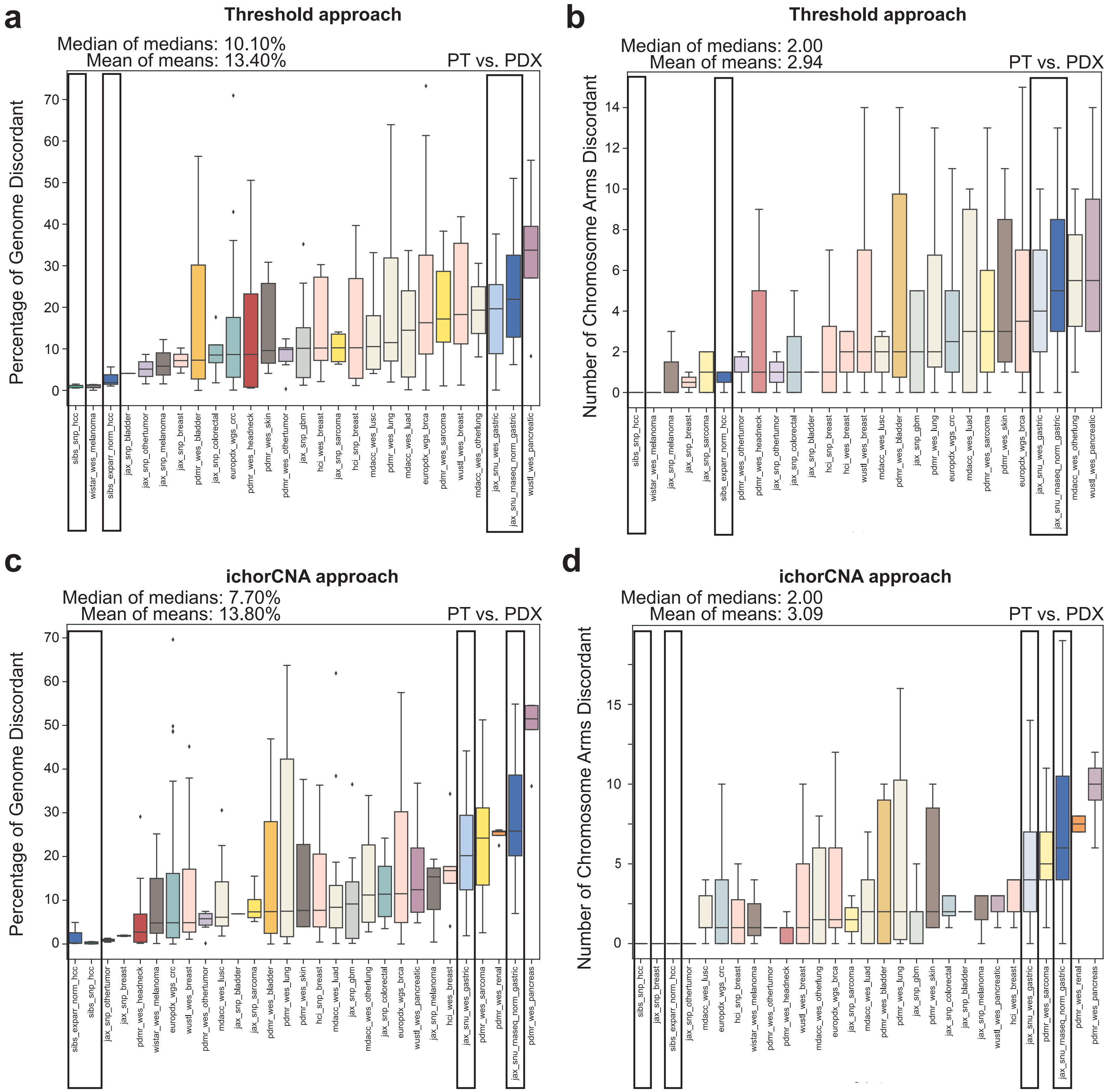

Supplementary Figure 6

Supplementary Figure 6: Discordance analysis with RNA-based copy number calls. (**a**) A thresholds-based comparison of the percent of the genome that is discordant between matched PT-PDX samples, including HCC and gastric cohorts assessed by RNA expression data. Note that the discordance level observed by RNA is highly similar to that assessed by DNA in the same cohorts (black triangles). (**b**) A thresholds-based comparison of the number of chromosome arms that are discordant between matched PT-PDX samples, including HCC and gastric cohorts assessed by RNA expression data. Note that the discordance level observed by RNA is highly similar to that assessed by DNA in the same cohorts (black triangles). (**c**) An ichorCNA-based comparison of the percent of the genome that is discordant between matched PT-PDX samples, including HCC and gastric cohorts assessed by RNA expression data. Note that the discordance level observed by RNA is highly similar to that assessed by DNA in the same cohorts (black triangles). (**d**) An ichorCNA-based comparison of the number of chromosome arms that are discordant between matched PT-PDX samples, including HCC and gastric cohorts assessed by RNA expression data. Note that the discordance level observed by RNA is highly similar to that assessed by DNA in the same cohorts (black triangles). Bar, median; colored rectangle, 25th to 75th percentile; whiskers, Q1 – 1.5*IQR to Q3 + 1.5*IQR; outliers were excluded from the plot.

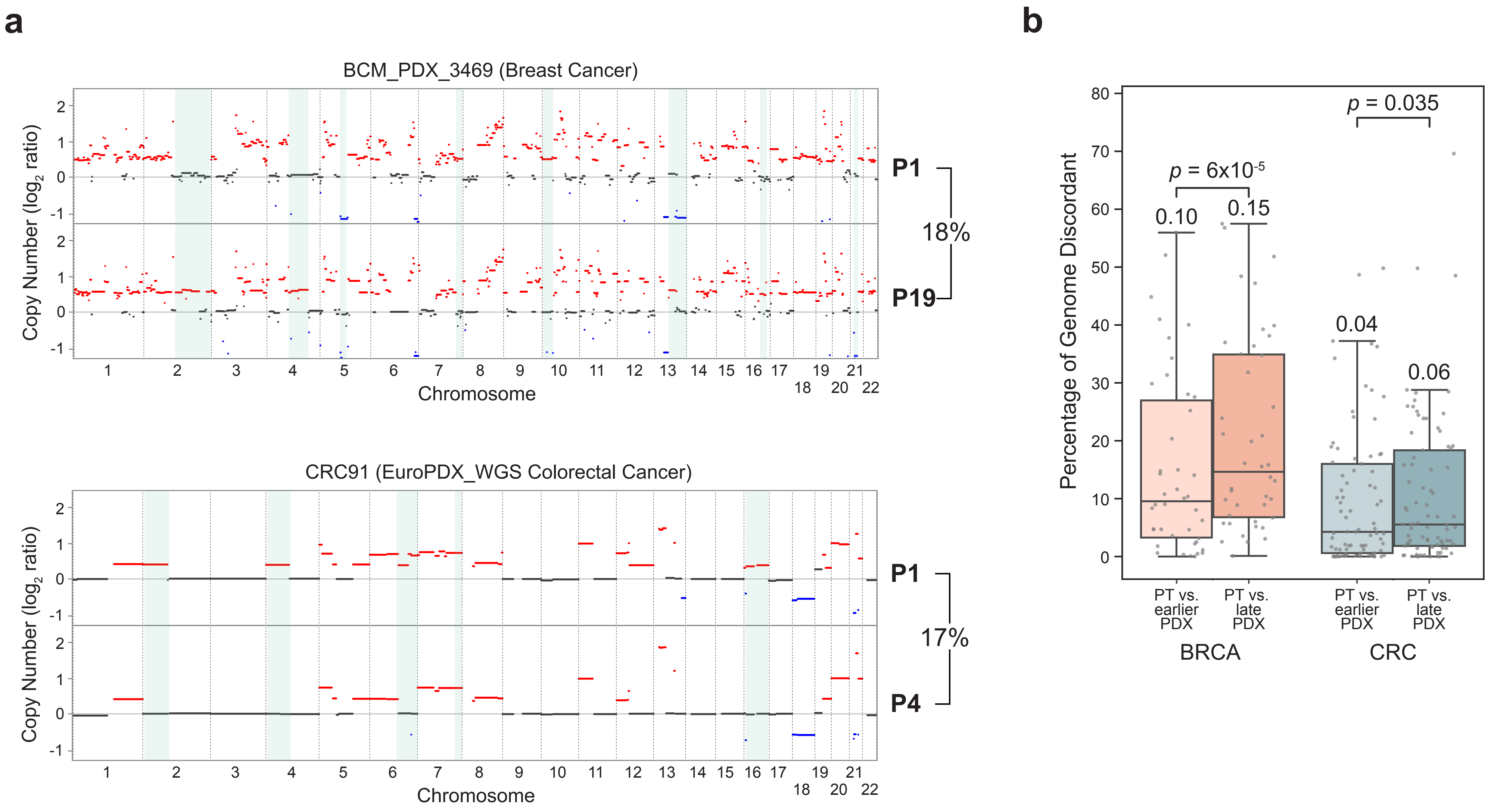

Supplementary Figure 7

Supplementary Figure 7: ichorCNA-based comparison of the copy number landscapes of PTs and PDXs (continued). (**a**) Examples of the CN differences between matched early passage PDX and late passage PDX samples: BCM_PDX (3469) cohort between P1 and P19 and EuroPDX_WGS_CRC (CRC91) cohort between P1 and P4. Red, CN gain; blue, CN loss. Prominent differences are highlighted with a light blue background. The fraction of the genome that is altered between samples is shown to the right of the plot. (**b**) A comparison of the percent of the genome that is discordant between PTs vs. earlier-passage PDXs and PTs vs. later-passage PDXs, in breast and colorectal cancer cohorts that included matched 'trios' of PT and PDXs from two passages. P-values obtained by a one-sided Wilcoxon signed-rank test. Circles, individual pairs; bar, median; colored rectangle, 25th to 75th percentile; whiskers, Q1 – 1.5*IQR to Q3 + 1.5*IQR.

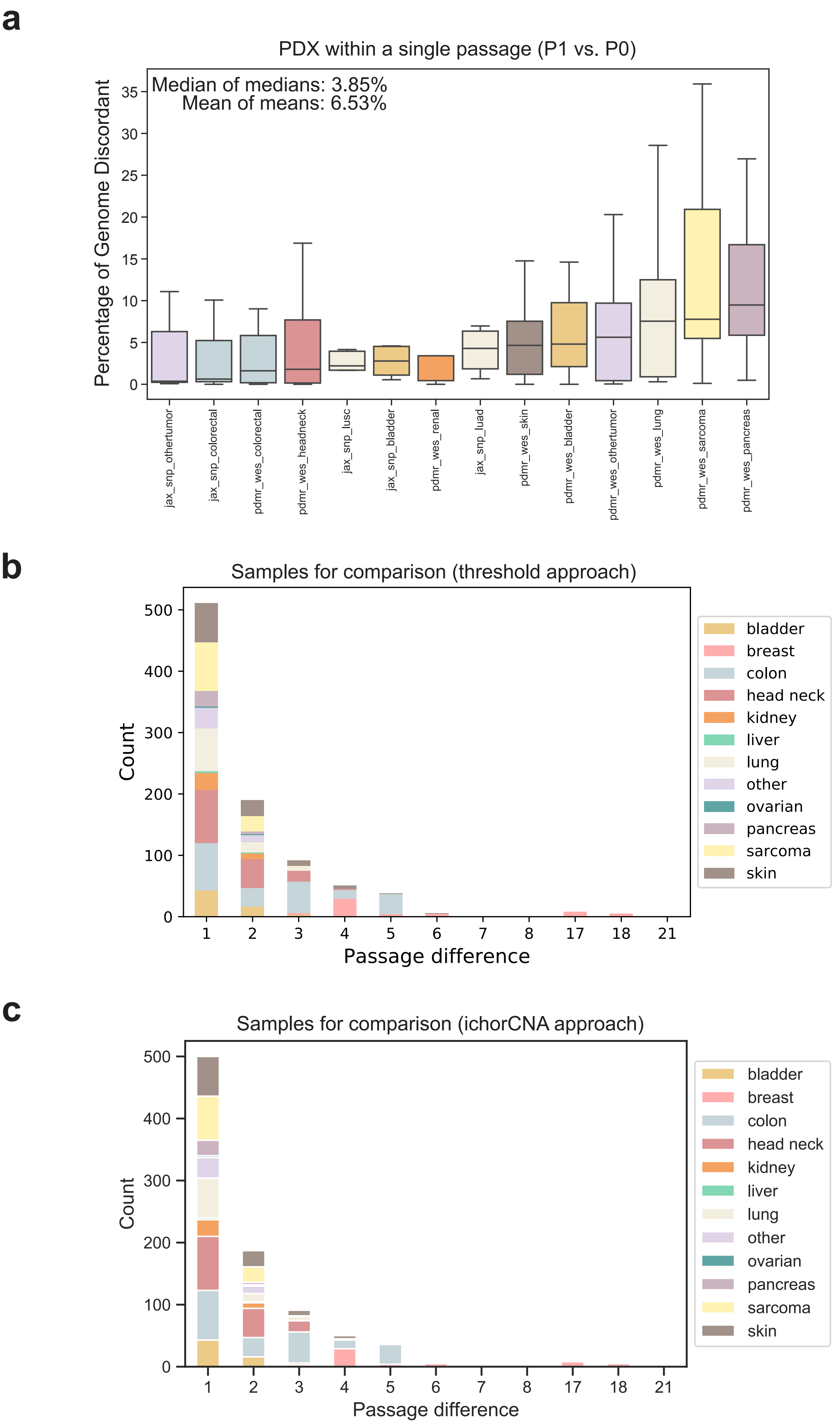

Supplementary Figure 8

Supplementary Figure 8: Comparison of the fraction of the genome that is altered within a single passage. (**a**) A cross-cohort comparison of the percent of the genome that is discordant between matched samples of PDXs at passage 0 and passage 1 with the ichorCNA-based analysis. In the median cohort, a median of 3.85% of the genome is altered between P0 and P1. Bar, median; colored rectangle, 25th to 75th percentile; whiskers, Q1 – 1.5*IQR to Q3 + 1.5*IQR; outliers were excluded from the plot. (**b**) The distribution of the passage difference between matched PDX samples across the Woo et al. cohorts, which were used for the thresholds-based analysis. (**c**) The distribution of the passage difference between matched PDX samples across the Woo et al. cohorts, which were used for the ichorCNA-based analysis.

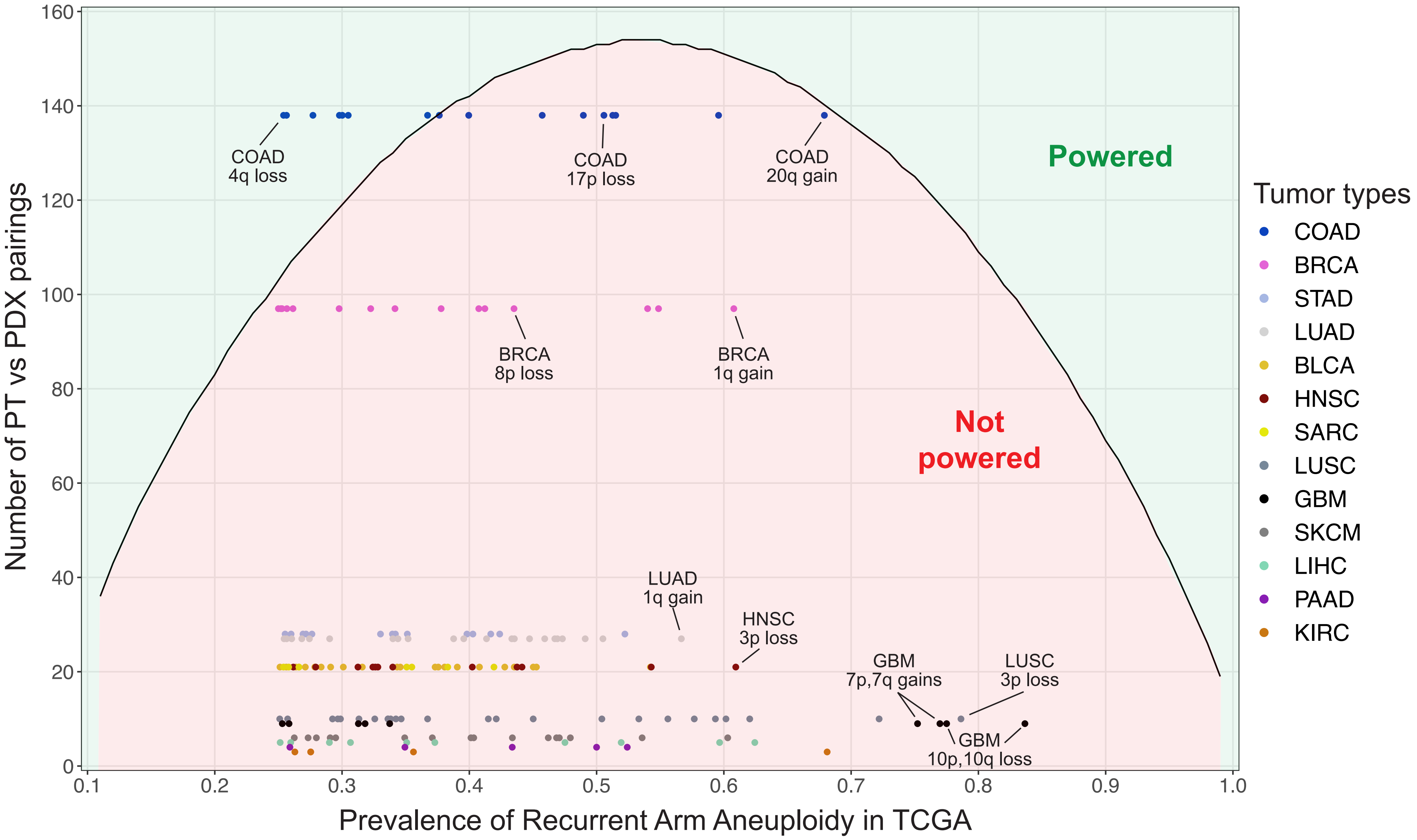

Supplementary Figure 9

Supplementary Figure 9: Power analysis to determine the required sample size to detect selection against recurrently altered chromosome arms. Chromosome-arm aneuploidy prevalence of TCGA tumors are shown as points for 13 tumor types (x-axis); recurrent arm-level aneuploidies with prevalence >= 0.25 are shown. The observed sample sizes of the corresponding tumor types in the Woo et al. study are indicated by the y-axis. Points above the black curve (green shading) are statistically powered to detect a 10% decrease in absolute prevalence from PTs to PDXs (with power of 80% and α=0.05), while points below the curve (red shading) are not. Statistical power analysis was performed using a single-sample, one-sided proportion test (**Methods**). Select recurrently altered chromosome arms are labeled.

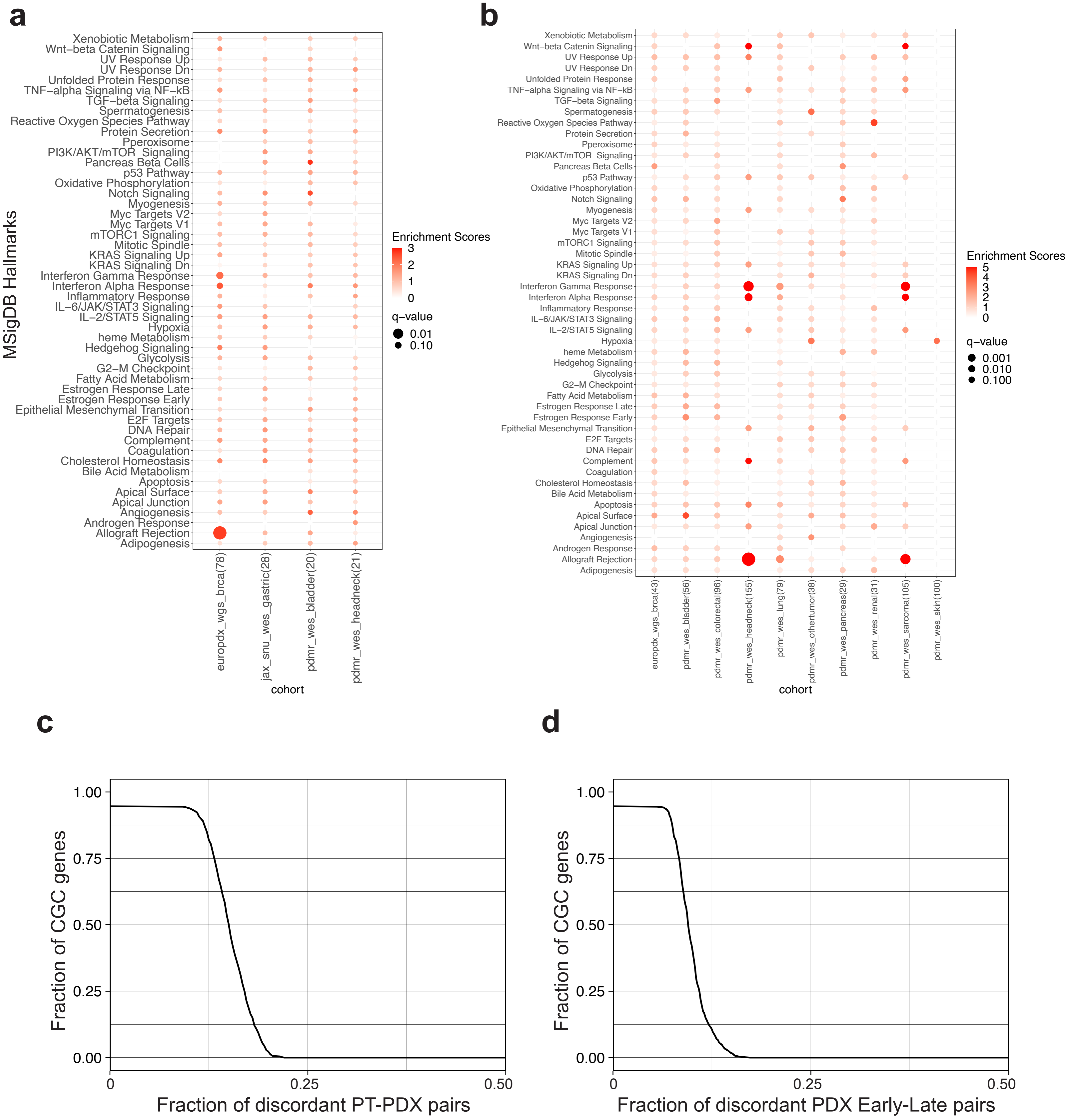

Supplementary Figure 10

Supplementary Figure 10: Gene-level analysis of recurrently discordant regions. (**a,b**) Heatmap presenting a gene set over-representation analysis of the genes that reside within CNAs that are recurrently differential between **(a)** PTs and PDXs and **(b**) earlier- and later-passage PDX samples. Enrichments were tested against the 50 MSigDB 'Hallmark' gene sets using a hypergeometric test. P-values were adjusted using Benjamini-Hochberg correction (q-values). Only cohorts having ≥ 20 PT-PDX or early-late sample pairs were included in the analysis. (**c**) Pancancer reverse estimator of cumulative distribution function (1 - eCDF) plot showing the fraction of tumors in which over a given number of COSMIC cancer genes is discordant between PTs and PDXs. Only 17 cohorts with ≥ 5 PT-PDX pairs were considered in this analysis. (**d**) Pancancer reverse estimator of cumulative distribution function (1 - eCDF) plot showing the fraction of tumors in which over a given number of COSMIC cancer genes is discordant between earlier and later PDX samples. Only 20 cohorts with ≥ 5 early-late pairs were considered in this analysis.

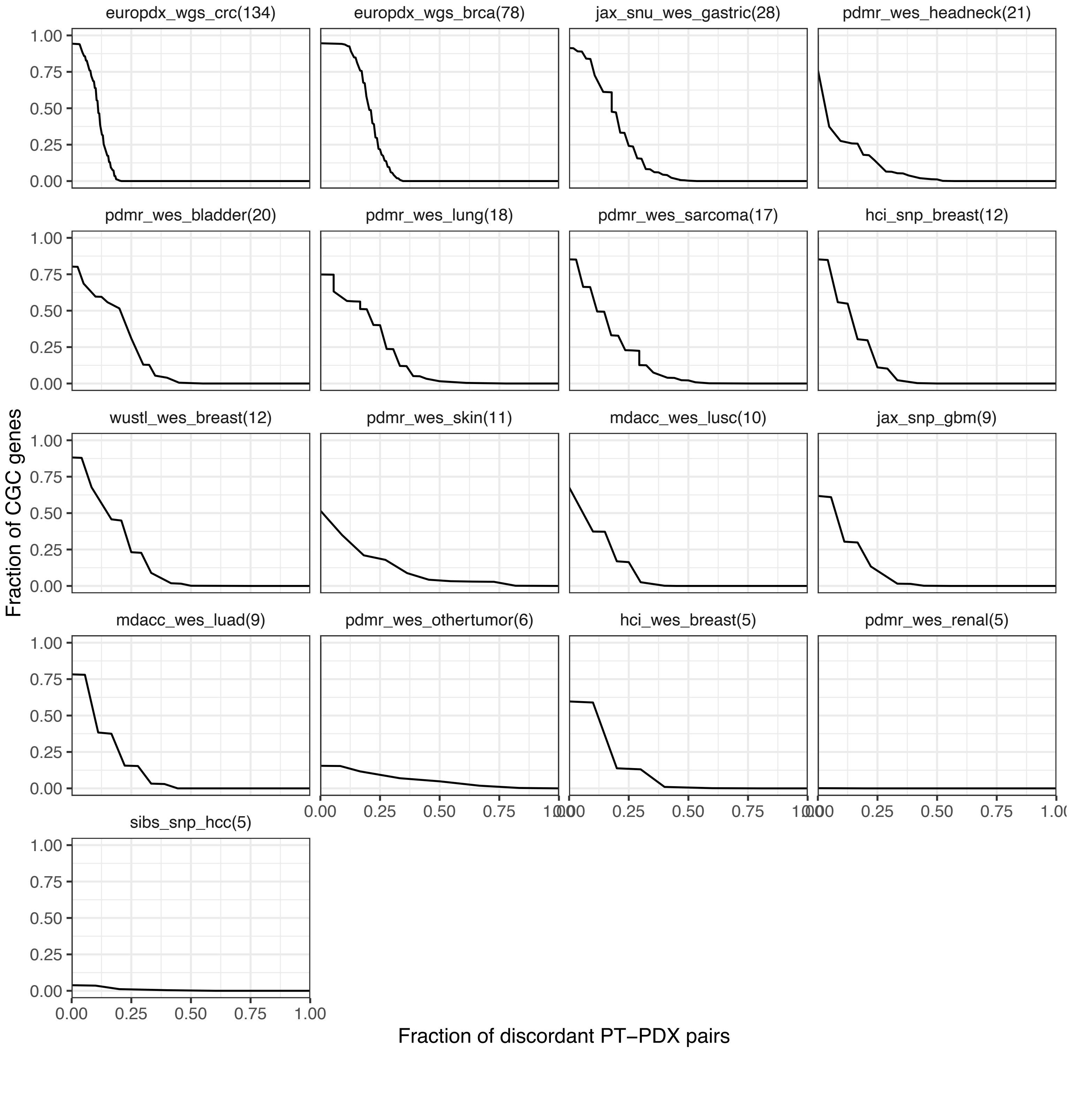

Supplementary Figure 11

Supplementary Figure 11: Analysis of cancer genes that are recurrently discordant between PTs and PDXs. Cohort-specific reverse estimator of cumulative distribution function (1 - eCDF) plot showing the fraction of tumors in which over a given number of COSMIC Cancer Gene Census (CGC) genes is discordant. Shown are cohorts with ≥ 5 PT-PDX pairs. A total of 704 CGC genes were considered in this analysis.

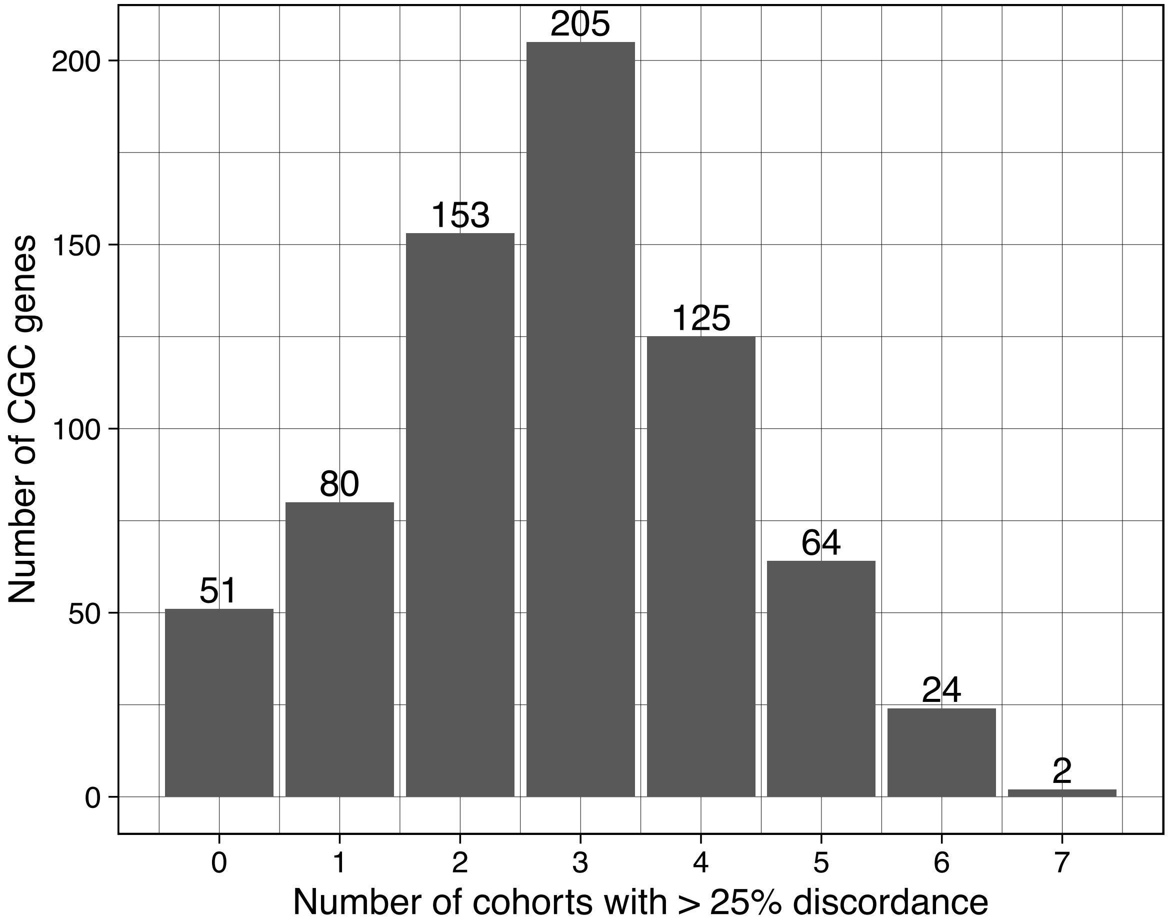

Supplementary Figure 12

Supplementary Figure 12: Distribution of cancer genes by the number of tumor cohorts in which they were recurrently discordant. COSMIC Cancer Gene Census (CGC) genes were counted if they were discordant in >25% of PT-PDX pairs for a given tumor cohort. Shown is the distribution of genes based on the number of cohorts in which the genes were observed to be discordant. 90 genes were discordant in > 25% of the PT-PDX pairs in ≥ 5 tumor cohorts. Only 17 cohorts with ≥ 5 PT-PDX pairs were considered. A total of 704 CGC genes were considered in this analysis.

Supplementary Data Legends

Supplementary Data 1: Thresholds-based CN discordance values. Included are all pairs that met the quality control requirements (**Methods**) and were used for the thresholds-based discordance analyses.

Supplementary Data 2: ichorCNA-based CN discordance values. Included are all pairs that met the quality control requirements (**Methods**) and were used for the ichorCNA-based discordance analyses.

Supplementary Data 3: Gene set over-representation analysis of genes within CNAs that are recurrently discordant. Frequency of discordant (threshold-based approach) pairs for each tumor cohort for all protein-coding genes, based on the overlapping 1Mb bin for the PT-PDX and early-late PDX comparisons. Gene set over-representation analyses are provided for all cohorts having ≥ 20 samples pairs. P-values and Benjamini-Hochberg-adjusted q-values were generated by hypergeometric tests (**Methods**).

Supplementary Data 4: Analysis of COSMIC Cancer Gene Census cancer genes that are recurrently discordant between PTs and PDXs. Frequency of discordant (threshold-based approach) pairs for each tumor cohort for 704 COSMIC Cancer Gene Census genes based on the overlapping 1Mb bin for the PT-PDX and early-late PDX comparisons. Counts of cohorts were based on genes having > 25% discordance frequency in cohorts with ≥ 5 total pairs. 17 cohorts with ≥ 5 PT-PDX pairs and 20 cohorts with ≥ 5 early-late pairs were considered.
