# Supplementary material for: DNA-based copy number analysis confirms genomic evolution of PDX models
Source: NPJ Precis Oncol. 2022 Apr 28;6:30. doi: 10.1038/s41698-022-00268-6 (PMC9050710; doi:10.1038/s41698-022-00268-6)
Supplement: Supplementary file 6 — REPORTING SUMMARY [file 41698_2022_268_MOESM6_ESM.pdf]

nature research

Corresponding author(s): Uri Ben-David

Last updated by author(s): Feb 15, 2022

Reporting Summary

Nature Research wishes to improve the reproducibility of the work that we publish. This form provides structure for consistency and transparency in reporting. For further information on Nature Research policies, see our Editorial Policies and the Editorial Policy Checklist.

Statistics

For all statistical analyses, confirm that the following items are present in the figure legend, table legend, main text, or Methods section.

n/a	Confirmed	
☐	☒	The exact sample size (*n*) for each experimental group/condition, given as a discrete number and unit of measurement
☐	☒	A statement on whether measurements were taken from distinct samples or whether the same sample was measured repeatedly
☐	☒	The statistical test(s) used AND whether they are one- or two-sided<br>*Only common tests should be described solely by name; describe more complex techniques in the Methods section.*
☒	☐	A description of all covariates tested
☒	☐	A description of any assumptions or corrections, such as tests of normality and adjustment for multiple comparisons
☐	☒	A full description of the statistical parameters including central tendency (e.g. means) or other basic estimates (e.g. regression coefficient) AND variation (e.g. standard deviation) or associated estimates of uncertainty (e.g. confidence intervals)
☐	☒	For null hypothesis testing, the test statistic (e.g. *F*, *t*, *r*) with confidence intervals, effect sizes, degrees of freedom and *P* value noted<br>*Give P values as exact values whenever suitable.*
☒	☐	For Bayesian analysis, information on the choice of priors and Markov chain Monte Carlo settings
☒	☐	For hierarchical and complex designs, identification of the appropriate level for tests and full reporting of outcomes
☐	☒	Estimates of effect sizes (e.g. Cohen's *d*, Pearson's *r*), indicating how they were calculated

Our web collection on statistics for biologists contains articles on many of the points above.

Software and code

Policy information about availability of computer code

Data collection	No software was used to collect this data.
Data analysis	Python 3.7, including os, matplotlib, re, statistics, glob, math, seaborn, and scipy.<br>R 3.6, including tidyverse, ggplot2, scales, data.table, foreach, plyr, doMC, GenomicRanges, and stringr.<br>Customized version of ichorCNA algorithm (copy number alteration prediction and purity and ploidy estimation): https://github.com/GavinHaLab/pdx_evolution_study/ichor_run.<br>All scripts used in this study can be found at https://github.com/GavinHaLab/pdx_evolution_study.

For manuscripts utilizing custom algorithms or software that are central to the research but not yet described in published literature, software must be made available to editors and reviewers. We strongly encourage code deposition in a community repository (e.g. GitHub). See the Nature Research guidelines for submitting code & software for further information.

Data

Policy information about availability of data

All manuscripts must include a data availability statement. This statement should provide the following information, where applicable:

- Accession codes, unique identifiers, or web links for publicly available datasets
- A list of figures that have associated raw data
- A description of any restrictions on data availability

The copy number profiles analyzed in this study are available in Supplementary Data I of Woo et al., https://doi.org/10.1101/861393.

Field-specific reporting

Please select the one below that is the best fit for your research. If you are not sure, read the appropriate sections before making your selection.

☒ Life sciences ☐ Behavioural & social sciences ☐ Ecological, evolutionary & environmental sciences

For a reference copy of the document with all sections, see nature.com/documents/nr-reporting-summary-flat.pdf

Life sciences study design

All studies must disclose on these points even when the disclosure is negative.

Sample size	No statistical methods were used to predetermine sample size.
Data exclusions	Samples whose copy number profiles had been estimated from RNA-sequencing and gene expression microarray data were excluded to avoid potential issues with expression-based copy number inference. With the thresholding approach for calling copy number, a sample was excluded from analysis if <5% of the 1 Mb bins in its genome with log2(CN ratio) values had absolute values of >= 0.3. With the ichorCNA approach for calling copy number, a sample was excluded from analysis if the sample contained <5% estimated tumor fraction and/or if <5% of the bins were clonally altered from neutral copy number after ploidy-adjustment and rounding.
Replication	Our findings were not replicated. We aimed to verify the reproducibility of our findings by analyzing copy number using two distinct methods: a thresholding approach and with ichorCNA.
Randomization	Randomization was not relevant to our study, as we were not testing different experimental conditions.
Blinding	Blinding was not relevant to our study, as we were not testing different experimental conditions.

Reporting for specific materials, systems and methods

We require information from authors about some types of materials, experimental systems and methods used in many studies. Here, indicate whether each material, system or method listed is relevant to your study. If you are not sure if a list item applies to your research, read the appropriate section before selecting a response.

Materials & experimental systems

n/a	Involved in the study	
☒	☐	Antibodies
☒	☐	Eukaryotic cell lines
☒	☐	Palaeontology and archaeology
☐	☒	Animals and other organisms
☐	☒	Human research participants
☒	☐	Clinical data
☒	☐	Dual use research of concern

Methods

n/a	Involved in the study	
☒	☐	ChIP-seq
☒	☐	Flow cytometry
☒	☐	MRI-based neuroimaging

Animals and other organisms

Policy information about studies involving animals; ARRIVE guidelines recommended for reporting animal research

Laboratory animals	Laboratory animal information as previously reported in Woo et al., https://doi.org/10.1101/861393.
Wild animals	The study did not involve wild animals.
Field-collected samples	The study did not involve field-collected samples.
Ethics oversight	N/A

Note that full information on the approval of the study protocol must also be provided in the manuscript.

Human research participants

Policy information about studies involving human research participants

Population characteristics	Human research participant information as previously reported in Woo et al., https://doi.org/10.1101/861393.
Recruitment	N/A

Ethics oversight | N/A

Note that full information on the approval of the study protocol must also be provided in the manuscript.
